# Simulation and performance analysis of plasma switch for microwave amplification in T shaped circular waveguide in the S band

**DOI:** 10.1038/s41598-025-04544-w

**Published:** 2025-07-01

**Authors:** Javad Taghinejad, Maede Rahmani, Ali Reza Niknam

**Affiliations:** Laser and Plasma Research Institute, Shahid Beheshti University, Tehran, 1983969411 Iran

**Keywords:** Microwave amplification, Plasma switch, High-power waves, T-shaped circular waveguide, CST simulation, Finite difference time domain (FDTD), Optics and photonics, Physics

## Abstract

In this work, the control and amplification of microwaves in a T-shaped circular waveguide in the S-band using a plasma switch are simulated using Computer Simulation Technology (CST) software and the Finite-Difference Time-Domain (FDTD) method. We present an innovative configuration that integrates a plasma switch into a T-shaped circular waveguide, enabling real-time control of plasma parameters to enhance microwave reflection and amplification. We determine the ratio of RF frequency to argon plasma frequency in the collisional regime and investigate the interaction, resonance, and complete reflection of electromagnetic waves. The variations in wave amplitude at three ports (input, side, and output arms) after interaction with the plasma switch at different plasma densities are analyzed and compared. Additionally, we discuss the temporal evolution of the electric field within the waveguide at the moment of complete reflection. By optimizing physical parameters, the proposed configuration demonstrates effective control, reflection, and amplification of high-power waves at high repetition rates.

## Introduction

Plasma switches are widely used in telecommunications and various industries due to their exceptional ability to manipulate microwave propagation. These devices offer high-speed operation, efficient waveguide isolation, and complete wave reflection, making them well-suited for high-frequency switching applications^1^. Plasma switches play a crucial role in microwave systems, RF pulse compression systems, medical technology, and wireless communications by enabling rapid modulation of wave paths. This capability significantly enhances waveguide system performance, improving wave control, facilitating amplification through constructive interference, and enabling precise wave path rotation^2,3^.

Compared to mechanical and electronic switches (such as transistors), plasma switches overcome limitations such as time delays, complex heat and pressure management, and dependence on mechanical components^4^. These issues can degrade system performance and efficiency, particularly in applications requiring rapid wave path adjustments or handling high-power waves, where electronic switches may be insufficient^4,6^. With their high reflectivity and short response times, plasma switches substantially enhance overall system efficiency. Additionally, using a circular waveguide instead of a rectangular one can extend the power range within specific frequency bands^7^. Plasma-based microwave switches are particularly well-suited for high-power radar systems, reconfigurable antenna arrays, RF pulse compression networks, and satellite communication platforms-applications where fast, high-power, and dynamically tunable signal control is critical.

Plasma switches are recognized as a significant innovation in microwave and telecommunications technologies^8,10^. The initial concepts and research in this area were introduced in the 1960s by researchers such as Gould and co-workers^5^. Their studies laid the foundation for advancements in plasma switch technology. Over the years, advancements in materials and manufacturing techniques have played a key role in the progression of this technology^6,10^. Today, plasma switches are widely applied in high-tech telecommunications, space, and military systems. Numerous experimental studies have confirmed the performance and reliability of plasma switches^12,15^. Semnani and colleagues were among the first to introduce microwave switches based on plasma within resonant cavities^10^. Two years later, Semnani and his team conducted experiments that explored the dimensional analysis and power reflection capabilities of plasma switches. They developed a dual plasma switch model with an absorbing mechanism^11^. Hussain et al. at 2024 presents the wave propagation analysis due to the interaction between electromagnetic waves and a finite-width slit embedded in an anisotropic medium^16^. The combination of plasma physics and the properties of electromagnetic waves provides a new perspective for industrialists to control and amplify RF waves for various applications^17,20^.

Solid-state and dielectric-based switches have been widely employed in RF and microwave systems due to their compact form factor and integration capabilities. For instance, PIN diodes and MEMS-based switches are frequently used for high-frequency signal routing and control^22–24^. However, these technologies often face limitations under high-power conditions, including thermal degradation, limited switching speed, and reduced tunability^24^. Recent advances have explored alternative switching mechanisms, such as plasma-based systems, which can overcome some of these challenges through dynamic tunability and robust high-power handling. In this context, the present work introduces a novel plasma-based switch integrated into a T-shaped circular waveguide, enabling fast ($<100\,\hbox {ns}$) and efficient RF control through plasma-wave interaction mechanisms.

This study presents the design, implementation, and optimization of a plasma switch operating in the S-band, aimed at controlling and amplifying microwave signals. The analysis focuses on critical physical parameters-including plasma density, electron frequency, collision frequency, and waveguide geometry-that influence wave reflection and constructive interference within the waveguide structure. At the plasma switch location, incident microwave signals are partially reflected, resulting in constructive interference and enhanced signal propagation toward the output port. The extent of reflection is closely linked to plasma properties such as skin depth and the frequency ratio between the microwave signal and plasma electrons, which determine resonance and maximum reflection conditions. A comparative study using argon plasma at three distinct densities demonstrates that the system can achieve up to a $1.4\times$ increase in output amplitude, with no signal reflection to the input port at the resonance frequency of 2.86 GHz. To accurately model the RF-plasma interaction, a hybrid simulation approach is employed: CST software provides detailed electromagnetic modeling of the waveguide configuration, while the Finite-Difference Time-Domain (FDTD) method captures the temporal dynamics of wave-plasma interactions. Together, these tools provide a comprehensive framework for optimizing high-power microwave control and amplification using plasma-based techniques.

Finally, the performance of the plasma switch is evaluated through in-depth CST and FDTD simulations, analyzing S-parameters, energy storage dynamics, and waveform evolution at the waveguide ports.

## Simulation and methodology

This study simulates a T-shaped circular waveguide using CST software, incorporating a plasma switch in the side arm to block and reflect microwaves. The simulation begins by modeling the waveguide and defining the plasma properties, focusing on effective parameters such as plasma frequency, collision frequency, and ion mean free path. As the microwave enters the T-junction, the simulation tracks the splitting of the wave, with one component traveling through the side arm and the other continuing along the main arm. The side arm, containing the switch, acts as a controllable on/off switch for the microwave power. When the plasma switch is on, a high-density plasma is generated within the side arm. The plasma’s high electron density and conductivity significantly alter the impedance of the side arm, effectively reflecting most of the power back into the main waveguide. The reflected wave is then analyzed to ensure constructive interference with the wave propagating through the main arm, facilitating efficient transmission toward the output port. The simulation is designed to ensure that the plasma switch operates with minimal loss while reflecting a broad range of the incoming wave’s bandwidth. This performance is primarily influenced by the electron density in the plasma region, which determines the plasma’s behavior. At higher densities, the plasma behaves more like a conductor, enhancing its reflective properties and reducing losses.

Additionally, the FDTD method is employed to theoretically analyze the interactions between electromagnetic waves and the plasma inside the tube. This method provides detailed insights into the amplitude and phase changes of the outgoing wave, complementing the CST simulations. The combination of these approaches offers a more comprehensive understanding of wave behavior in plasma environments, which is crucial for optimizing the design and performance of plasma switches. All simulations were performed using CST Studio Suite 2022 (Dassault Systèmes). The software is available at: https://www.3ds.com/products-services/simulia/products/cst-studio-suite. The results of this study are expected to have significant implications for various applications, including array communication systems, filter banks, phase shifting in multiple systems, and radar technologies. The detailed methodology employed in this research is outlined in the following sections.

### Structure of simulation

**Fig. 1:**
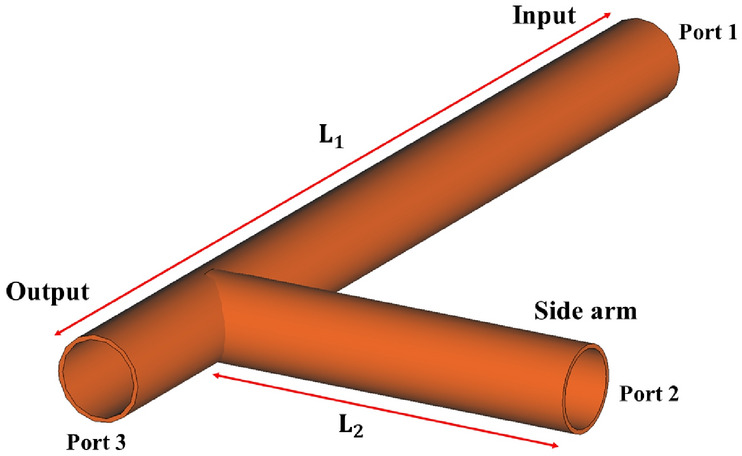
Schematic of a T-shaped circular waveguide in the S-band with three ports: input, side, and output.

The physical principle of plasma switch and T-junction waveguide described briefly below. A T-shaped circular waveguide, operating in the S-band, is designed with three ports: input, side, and output. The waveguide is used for impedance matching and efficient wave routing, with a plasma switch incorporated in the side arm. Figure 1 illustrates the overall schematic of the waveguide, which is modeled with 10 cells per wavelength for accurate simulation.

**Fig. 2:**
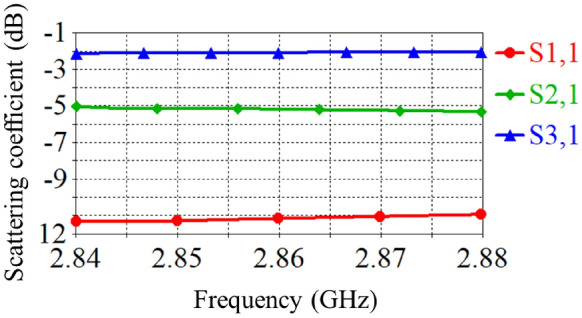
Simulation results of the S-parameters for the T-shaped circular waveguide with three ports: input port, side port, and output port.

A standard circular waveguide made of copper, with a radius of $a = 45$ mm, is used in the S-band frequency range of 2 GHz to 4 GHz. For the simulation, an operating frequency of $f = 2.86$ GHz is selected. The free-space wavelength corresponding to this frequency is $\lambda = {c}/{f} = 10.489$ cm, where *c* is the speed of light. The guided wavelength at this frequency is calculated using the following formula:

1 $$\begin{aligned} \lambda _{g}=\dfrac{\lambda }{\sqrt{1-\left( \dfrac{f_{c}}{f}\right) ^{2}}}, \end{aligned}$$

where $f_{c} = {p_{01}}/({2 \pi a \sqrt{\varepsilon _0 \mu _0}})$ is the cutoff frequency, with $\varepsilon _0$ and $\mu _0$ representing the permittivity and permeability of free space, respectively. This frequency can be calculated for the first mode of the circular waveguide, $TE_{01}$. Here, $p_{01}$ corresponds to the first root of the Bessel function, which for this mode is 2.405. The guided wavelength, which is critical for determining wave propagation or attenuation, is denoted by $\lambda _{g}$. The waveguide length is specified as $L_1 = 10 \times \lambda _{g}$, the side arm length as $L_2 = 2 \times \lambda _{g}$, and the height as $8 \times \lambda _{g}$. Using the Eq. 1 the guided wavelength determined as $11.46\,cm$ at the mentioned frequency (assuming air as the dielectric medium inside the waveguide). Moreover, we designate the S-parameters (scattering coefficient) of the input, side, and output ports as $S_{11}$, $S_{21}$, and $S_{31}$, respectively. A Gaussian excitation signal of an amplitude of 1 is input the waveguide from port 1 over the frequency range of 2.84 GHz to 2.88 GHz. The S-parameters of this waveguide are shown in Fig. 2.

As shown in Fig. 2, part of the incoming signal is reflected back to Port 1, while a significant portion travels through the side arm to Port 2. Additionally, a portion of the incoming pulse is transmitted through the waveguide and reaches Port 3, which serves as the output. When the plasma switch is activated, the wave path at the switch location is modified due to the short-circuiting effects. However, when a minimal voltage is applied to the plasma switch, the generated plasma effectively blocks the wave path in the side arm, resulting in the reflection of the wave.

**Fig. 3:**
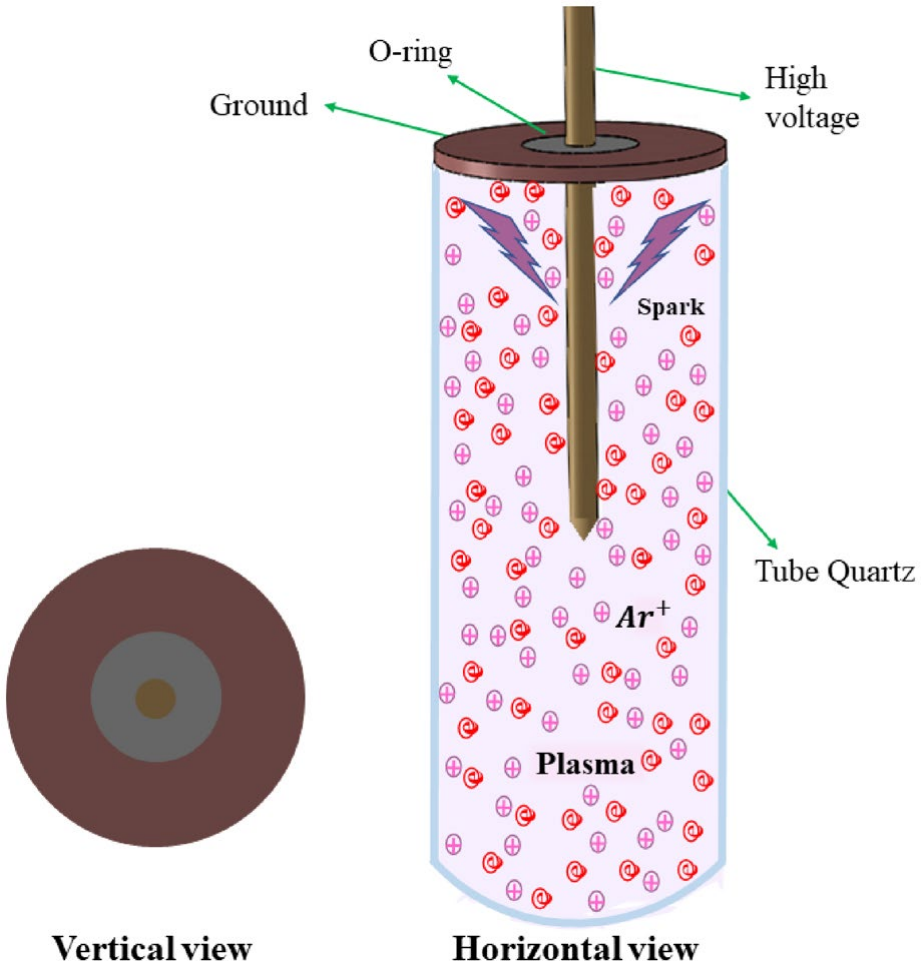
Diagram of plasma switches used for scattering of electromagnetic waves.

The plasma switch is located at a position of ${3}/{4}\,\lambda _{g}$ along the side arm. This positioning maximizes the interaction between the wave field and the plasma, thereby enhancing wave reflection. The end of the switch is grounded and contains a metallic rod with a voltage applied between the rod and an insulator ring. The potential difference between the ground and the metallic rod generates a plasma spark. To prevent the dispersion of plasma ions within the waveguide and maintain a stable plasma density, a quartz tube is used with $7\,mm$ radius. Quartz is selected for its excellent dimensional stability, resistance to thermal shock, chemical durability during plasma formation, and high transparency to a wide range of electromagnetic waves, making it an ideal material for the tube. Figure 3 shows the structure of plasma switch. By carefully choosing the density, voltage, gas type, and precise design of the switch, we assume a uniform plasma within the tube, thus neglecting radial frequency variations due to changes in plasma density.

Plasma formation within the quartz tube is achieved solely by applying a high DC voltage (1 kV) between the internal metal rod and the grounded outer wall of the tube. This voltage initiates ionization of the argon gas, resulting in a stable plasma discharge. The microwave signal does not participate in initiating the plasma but instead interacts with the pre-formed plasma during RF operation. Therefore, no combined RF-DC discharge mechanism is considered in this study.

Impinging RF wave on the switch in the side arm begin to interacts with the plasma inside the quartz tube. The reflection or transmission parameter and also phase shift of microwave near the plasma switch varied by the ratio of the RF wave frequency to the plasma frequency. This parameter can be described using electrical parameters such as the dielectric constant $\varepsilon _p$ and conductivity $\sigma _p$. Both the dielectric constant and conductivity are directly vary by the electron density, applied voltage, and type of gas used in the switch.

In short, the plasma switch operates in two distinct functional states. In the off state, where plasma density is negligible, the system permits nearly lossless microwave transmission through the side port, with minimal reflection. In the on state, plasma generation leads to strong reflection of the incident signal, enabling dynamic wave redirection or constructive interference within the main waveguide. This bistable behavior supports efficient signal modulation and routing. In contrast to conventional solid-state switches-which may be limited by thermal constraints, mechanical wear, or slower switching times-the proposed plasma-based switch offers nanosecond-scale response time, high-power tolerance, and low insertion loss, making it highly suitable for demanding RF applications.

### CST simulation

The cross-sectional area for argon ion collisions can be calculated using the formula $\sigma _q = \pi r^2$, where $r = 96 \times 10^{-12} \, \text {m}$ is the argon ion radius. Hence, this parameter is calculate as $\sigma _q = 2.8938 \times 10^{-20} \text { m}^2$. This parameter helps to determine the collision rate and consequently the plasma mean free path. Equation 2 shows the mean free path for high-frequency plasma as a function of plasma density variations and can provide valuable information about the properties of the generated plasma.

2 $$\begin{aligned} \lambda _{i}=\dfrac{1}{\sqrt{2}n_{i}\sigma _{q}}, \end{aligned}$$

where, $n_i$ is the ion density. On the other hand, the collision frequency $\nu _m (= {v_i}/{\lambda _i})$ is determined by calculating the ratio of the ion velocity to the mean free path. Here, $v_i (= \sqrt{{2eV}/{M_i}})$ is the ion velocity, *e* is the electron charge, and $M_i = 6.63 \times 10^{-26} \text { kg}$ is the mass of the argon ion. Hence, altering the plasma density within the switch tube result in variation of collision frequencies, mean free paths, electrical conductivity, and ions mobility. These fundamental parameter changes result in different capabilities of the plasma to short-circuit the wave path. Consequently, different plasma density caused the different amount of reflection or transmit of RF wave from the plasma switch.

The electron density ($n_e$) and ion density in the spark plasma will be equal to the plasma density ($n_0$). In this case, the plasma electron frequency can be calculated as $\omega _{pe} = \sqrt{{n_e e^2}/{\varepsilon _0 m_e}}$, where $m_e$ is the electron mass. On the other hand, the electrical conductivity of the spark plasma can be calculated using the following relation:

3 $$\begin{aligned} \sigma _{p}=\dfrac{\omega _{pe}^{2}\varepsilon _{0}}{i\omega +\nu _{m}}, \end{aligned}$$

where $\omega = 2\pi f$ is the angular frequency of the microwave entering the waveguide, the effective dielectric constant of the plasma for the collisional case can be derived from the Drude model as follows:

4 $$\begin{aligned} \varepsilon _{p}=1-\dfrac{\omega _{p^{2}}}{\omega (\omega -i\nu _{m})}. \end{aligned}$$

For complete reflection of the input signal in the side arm, the pulse frequency must be lower than the plasma frequency. Source frequency $f = 2.86 \, \text {GHz}$ results the angular frequency of $\omega = 17.967 \, \text {GHz}$. Using suitable plasma switch radius, the dielectric constant $\varepsilon _P$ and also the amount of signal transmission or reflection by the plasma switch can be controlled by the plasma frequency. Plasma is generated using a fixed DC voltage of 1 kV, sufficient to initiate discharge within the tube. Different plasma densities, and their corresponding plasma parameters, are achieved by adjusting the gas input conditions and are summarized in Table 1.

For the case where $\omega < \omega _P$, which occurs for most microwave signal frequencies, $\varepsilon _P$ becomes negative. In plasma, the presence of electrons and ions creates an electrically active environment. These electrons and ions produce specific electrical properties that can lead to a negative dielectric constant. Additionally, because of electric and magnetic currents, the plasma’s response to electric and magnetic fields becomes more complicated. This frequency response can result in a negative dielectric constant, causing electromagnetic waves to be unable to propagate easily through the plasma and instead be reflected.

**Table 1:** Calculation of plasma characteristics for three different plasma densities, as presented in *a*, *b*, and *c*.

Cases	$n_0$ ($1/\hbox {m}^3$)	$\nu _m$ (1/s)	$\lambda _i$ (m)	$\omega _P$ (rad/s)
a	$1.3 \times 10^{20}$	$1.223 \times 10^{15}$	$0.568 \times 10^{-10}$	$6.428 \times 10^{11}$
b	$1.3 \times 10^{18}$	$1.223 \times 10^{13}$	$0.568 \times 10^{-8}$	$6.428 \times 10^{10}$
c	$1.3 \times 10^{16}$	$1.223 \times 10^{11}$	$0.568 \times 10^{-6}$	$6.428 \times 10^9$

The plasma dielectric constant can be determined by substituting $\omega _p$ and $\nu _m$ as functions of the input wave frequency and the argon ion density within the plasma bulk. The dielectric constant is then calculated using these parameters as follows:

5 $$\begin{aligned} \varepsilon _{p}=\varepsilon _{p_r}+\varepsilon _{p_i}=1-\dfrac{n_0e^{2}}{4\pi ^{2}f^2\varepsilon _{0}M_{e}\left( 1-i\sqrt{\frac{eV}{M_i}}\frac{n_i\sigma _q}{\pi f}\right) }. \end{aligned}$$

**Fig. 4:**
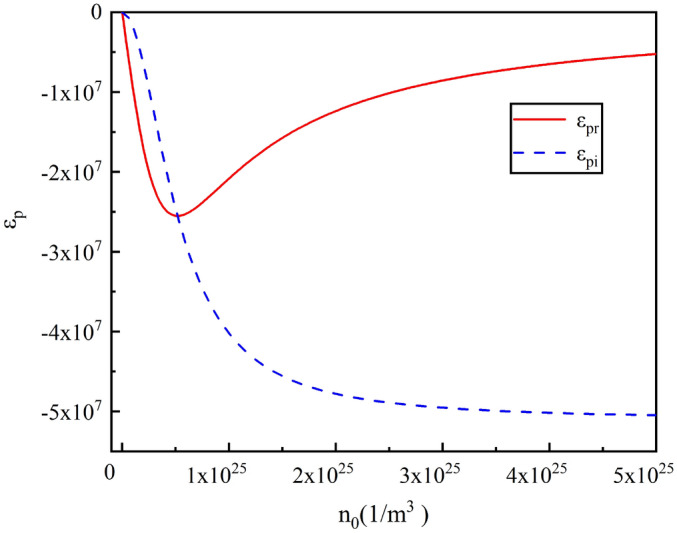
Variation of the real and imaginary parts of plasma permittivity within the plasma switch as a function of plasma density.

The plasma permittivity can be determine versus type of gas, particle density, and applied voltage. Figure 4 illustrate variation of the real and imaginary part of plasma permittivity versus plasma density for Ar plasmas and $f=2.86\,GHz$ and $V=1\,KV$.

According to Equation 5, in the plasma region, the permittivity $\varepsilon _p$ is complex and consists of a real part ($\varepsilon _{p_r}$) and an imaginary part ($\varepsilon _{p_i}$). These two parts describe the plasma’s response to electromagnetic fields. As plasma density increases, the plasma frequency increases, and the real part of the permittivity decreases. For a wave with a frequency lower than the plasma frequency, the plasma will have a refractive index that decreases with increasing electron density, causing the real part of permittivity to approach zero. This indicates the plasma becoming increasingly transparent to certain frequencies as the electron density rises.

Figure 4 illustrates that as plasma density increases, the real part of the plasma permittivity decreases and may even become more negative. Concurrently, as ion-neutral collisions increase, the imaginary part of the permittivity decreases due to more energy being lost in collisions, resulting in reduced energy absorption by the plasma.

With the increase in plasma density, the number of charged particles per unit volume also rises. This enhances electrical currents and results in more electromagnetic interactions between the RF wave and the plasma, transferring more energy to the plasma. This, in turn, increases the reflection of the wave from the plasma switch. Additionally, as plasma density increases, the speed of charge carriers rises, but their mobility decreases. Consequently, higher plasma density leads to greater wave reflection and reduced absorption by the plasma. These effects can be used to fine-tune the plasma switch’s properties, adjusting how the wave is refracted or absorbed.

When the wave frequency is close to the plasma frequency, amplification typically occurs. This is due to the plasma’s strong absorption of the electromagnetic waves, as electrons in the plasma region resonate strongly with the wave. This resonance enhances the absorption of wave energy by the plasma and also increases the plasma’s dielectric constant. Accurately determining the ratio of the microwave frequency to the plasma frequency is essential for predicting wave-plasma interactions.

However, since the wavelength of the microwave is much larger than the radius of the plasma switch tube ($\lambda \gg a$), the electric field near the plasma tube is approximately non-rotational ($\nabla \times \textbf{E} \approx 0$). In this case, the electric field distribution can be described by the gradient of a scalar potential ($\textbf{E} = -\nabla V$). The dielectric constant of the plasma is considered homogeneous, and thus the potential inside and outside the plasma tube satisfies Laplace’s equation with no variation along the *z*-axis. Therefore, the potential in a cylindrical tube is represented by the following linear combination:

6 $$\begin{aligned} \phi _{n}=\sum _{n=1}^{\infty }(A_{n}a^{n}+B_{n}a^{-n})(C_{n}\cos n\theta + D_{n} \sin n\theta ), \end{aligned}$$

where *n* is an integer. Using the zero potential condition at the center of the cylinder, the equality of the potential value at $\theta = 0$ and $\theta = \pi$ yields the following potential function inside the tube:

7 $$\begin{aligned} \Phi _{in}=\sum _{n=1}^{\infty }A_{n}a^{n} \sin n\theta . \end{aligned}$$

As the radius of the plasma tube approaches infinity ($a \rightarrow \infty$), the potential outside the tube asymptotically approaches the potential of the incident wave. Therefore, the potential in the external region is expressed as:

8 $$\begin{aligned} \Phi _{out}=\sum _{n}B_{n}a^{-n} \sin n\theta + E_{inc} a \sin n\theta , \end{aligned}$$

where $E_{inc}$ is the microwave field. At the boundary between the plasma and vacuum, the normal component of the displacement field ($\textbf{D} = \varepsilon _p \textbf{E}$) and the tangential electric field are equal across the two boundaries. By differentiating the potential and equating the potential inside and outside the tube, the amplitude for $n = 1$ is given by:

9 $$\begin{aligned} (1+\varepsilon _p)A_{1}=0. \end{aligned}$$

Therefore, in the case where $\varepsilon _p = -1$, the electric field within the plasma region is significantly enhanced and resonance occurs. Consequently, using equation 4, the microwave frequency required for resonance at a specific plasma frequency is given by:

10 $$\begin{aligned} \omega =\dfrac{2i\nu _{m}+\sqrt{2\omega _{p}^{2}-\nu _{m}^{2}}}{2}, \end{aligned}$$

where for collisionless plasma, $\nu _{m}$ is 0, so $\omega =\omega _{p}/\sqrt{2}$^21^. The Eq. 10 demonstrates that the needed microwave frequency of resonance and RF amplification is dependent to the plasma frequency, collision frequency, and consequently, to the plasma density, kind of plasma gas, and applied voltage. Hence, the plasma density and applied voltage of resonance and amplification of microwave can be determined. Typically, resonance occurs when the microwave frequency and plasma frequency are significantly different, which allows for effective interaction between the electromagnetic waves and the plasma, leading to increased wave energy and resonance. Conversely, when the microwave frequency and plasma frequency are close, resonance is minimal, and the waves tend to pass through the plasma without significant interaction.

As illustrated in the Fig. 4, when the ($\omega _p$) to be much larger than the incident wave frequency ($\omega _p\gg \omega$), the real part of dielectric constant of the plasma approaches zero. This condition, known as “zero dielectric constant,” results in the incoming electromagnetic waves being reflected rather than transmitted through the plasma. The mismatch between the wave frequency and plasma frequency leads to wave reflection due to the lack of interaction between the waves and the plasma.

**Fig. 5:**
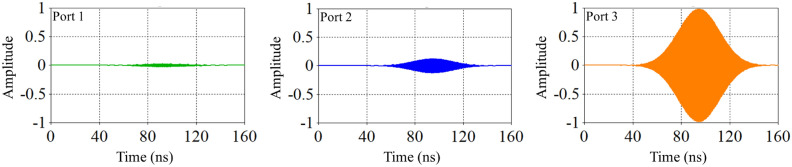
Comparison of pulse amplitudes at three different ports after switching electromagnetic wave for case (a).

**Fig. 6:**
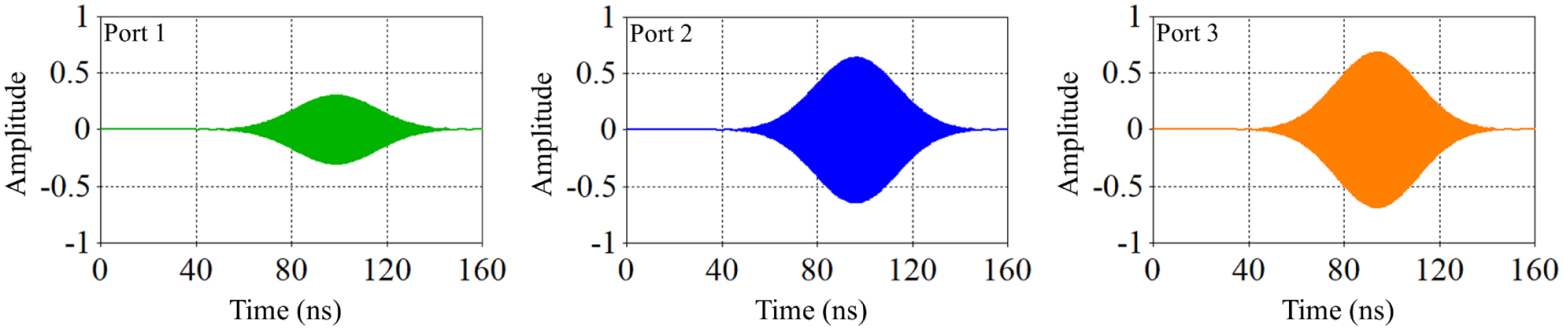
Comparison of pulse amplitudes at three different ports after switching electromagnetic wave for case (b).

**Fig. 7:**
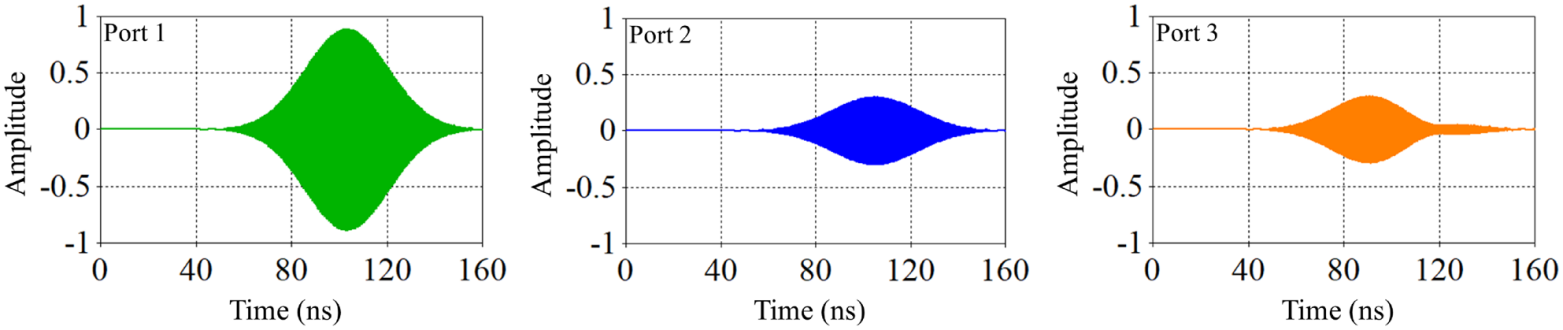
Comparison of pulse amplitudes at three different ports after switching electromagnetic wave for case (c).

When the plasma frequency is significantly higher than the wave frequency, the wavelength of the incoming electromagnetic waves is relatively short compared to the distance between plasma particles. Consequently, the waves cannot effectively interact with the plasma particles or transfer their energy to the plasma. This results in the waves passing through the plasma in a reflective manner, reducing the dielectric constant and leading to the “zero dielectric constant” phenomenon. Figures 5, 6 and 7 compares the amplitude of pulses arriving at three ports for three different cases presented in Table 1.

Comparing the output pulse (third port of all cases), it can be observed that as the plasma density inside the switch increases, the amplitude of the output pulse also increases. Conversely, at the second port, the pulse amplitude decreases. This is because the ability of switch to reflect microwave decreased, in which leading to an increase in the amount of pulse passing through the switch. Therefore, if increasing the plasma density does not completely block the wave path at the side wall, impedance matching occurs in such a way that the amount of reflected pulse to the input port also increases. If the amplitude of the reflected wave to the input port becomes excessively high, there is a risk of damaging the wave source.

According to Figures 5, 6, 7, the amplitude of the wave concentration within the waveguide reaches its maximum value up to 100 nanoseconds. This indicates that the energy stored inside the waveguide is at its peak at this time, but changes over time.

**Fig. 8:**
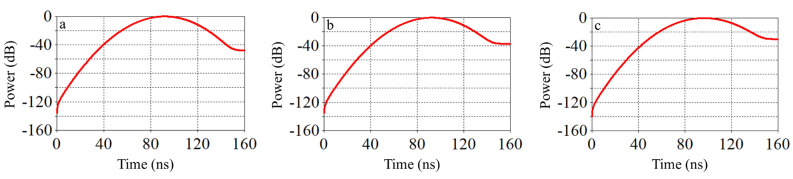
Comparison of power storage during switching the electromagnetic within the waveguide for three different cases presented in Table 1.

Figure 8 compares the temporal distribution of energy inside the waveguide for three different cases. This figure compare the amount of energy accumulated and subsequently dissipated by electromagnetic waves in the system for three different cases. In other words, this section illustrates the different concentration of electromagnetic energies within the system and provides temporal evolution of these cases. This information can be used for analyzing and optimizing electromagnetic systems and plasma switch characteristics. From this figure, it can be concluded that when the plasma switch fully removes the electromagnetic wave from the system, the energy is completely dissipated from the waveguide. As a result, the disturbance caused by the residual energy in the waveguide also approaches zero. In fact, when switching occurs more frequently, the disturbance caused by plasma formation from the residual energy is reduced, and plasma is generated in a timely manner without delay.

To investigate the behavior of electromagnetic waves with argon plasma at the switch location, it is necessary to estimate the interaction between the electromagnetic wave and the plasma. Numerical solution are used to analyses the interaction between the electromagnetic wave and the plasma is an important topic that will be addressed in detail in the following sections.

## FDTD numerical analysis

To conduct a comprehensive study of the T-shaped waveguide with a plasma switch, it is essential to examine the role of the plasma switch and wave reflector in greater detail. By numerically solving the electromagnetic wave interaction at the edge of the plasma switch, we can analyze that how the RF waves interact with the plasma switch in three distinct cases, labeled as a, b, and c, as presented in Table 1. To achieve this, the necessary parameters for the numerical solution of the interactions in this region are calculated and the grid parameters for this configuration are specified based on Yee’s algorithm.

We select $n_E = 200$ grids with integer coordinates for the electric field and $n_H = 199$ grids with half-integer coordinates for the magnetic field. Considering the wave speed and spatial distance, the total computation time, $T_{max}$, is determined. For the one-dimensional numerical solution, the plasma switch is assumed to have a circular cross-section, however we use a circle with known material properties such as dielectric constant, electrical conductivity, and magnetic permeability. Given the high frequency of the RF wave, the wavelength and guided wavelength are computed, with the spatial step defined as $\Delta x = \lambda _g / 10$ and the time step as $\Delta t = \Delta x / C$. A circular cross-section with a radius of 7 mm is defined, with argon plasma material placed in the center of the waveguide.

**Fig. 9:**
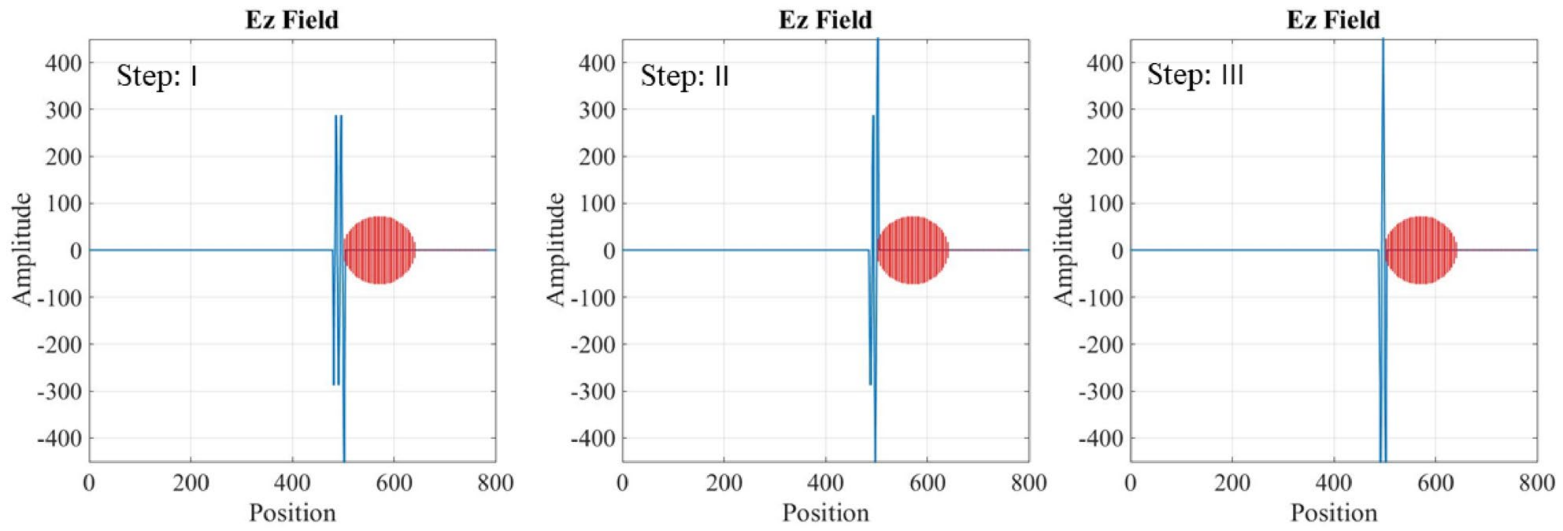
Temporal variation of the RF wave during interaction with the plasma tube for case (a), Step: I is for impinging RF wave, Step: II is for Rf Wave-plasma start to interaction, Step III: is for interacted RF wave-plasma.

**Fig. 10:**
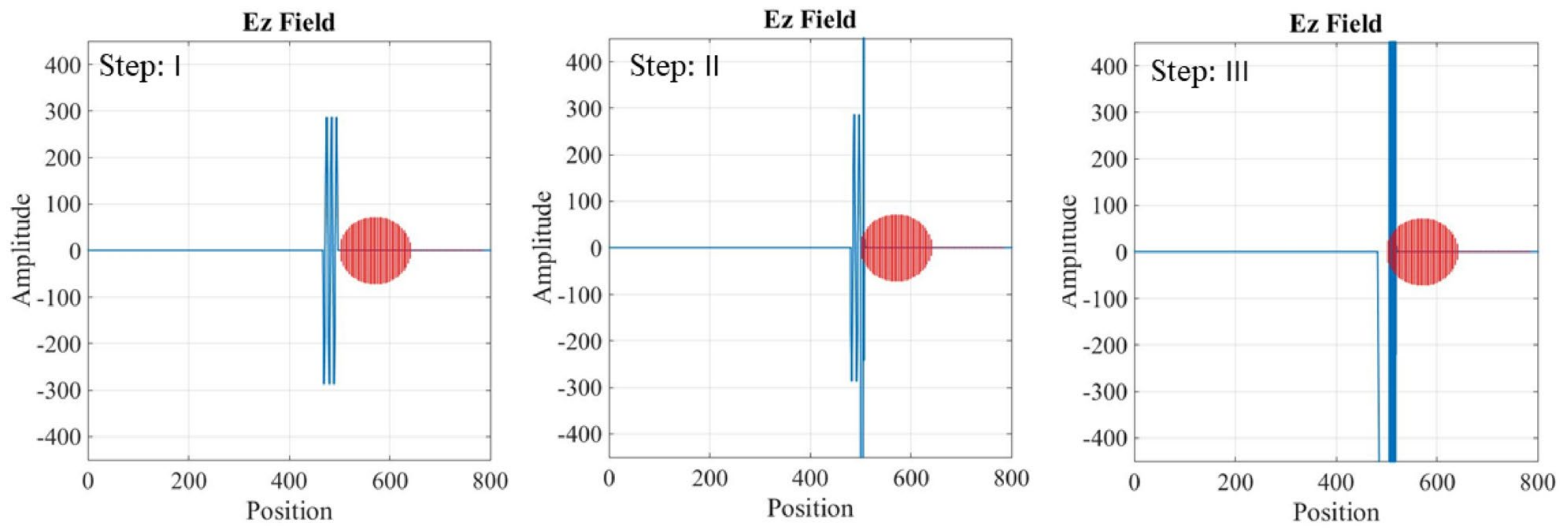
Temporal variation of the RF wave during interaction with the plasma tube for case (b), Step: I is for impinging RF wave, Step: II is for RF Wave-plasma start to interaction, Step III: is for interacted RF wave-plasma.

**Fig. 11:**
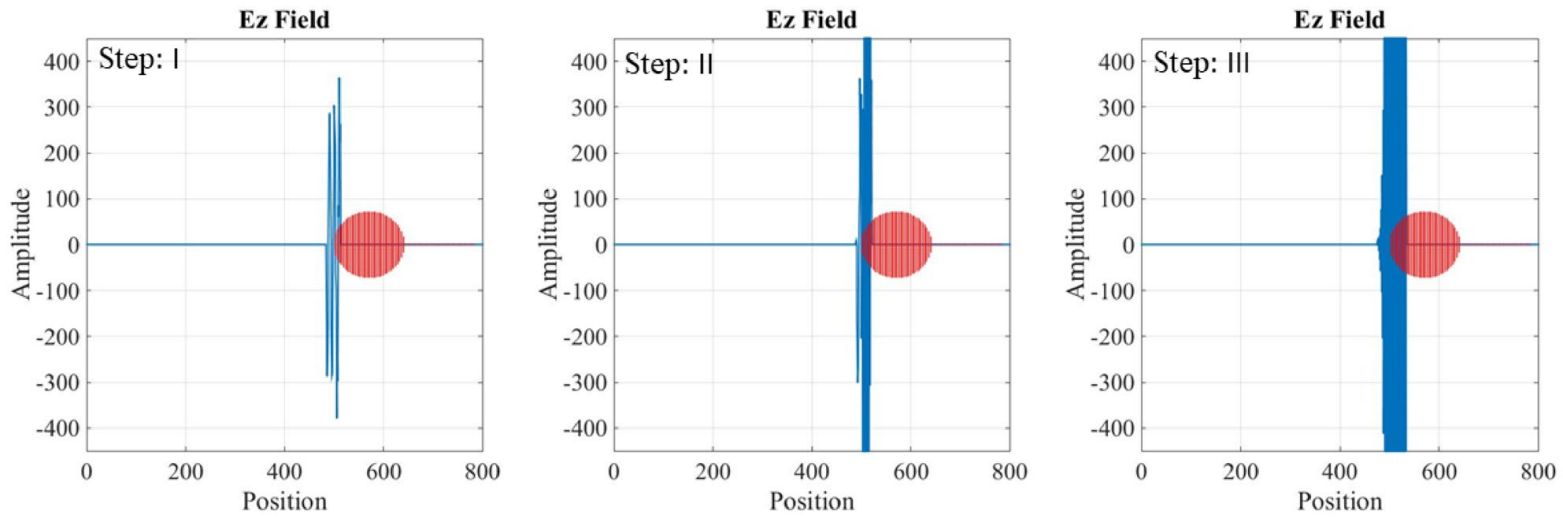
Temporal variation of the RF wave during interaction with the plasma tube for case (c), Step: I is for impinging RF wave, Step: II is for Rf Wave-plasma start to interaction, Step III: is for interacted RF wave-plasma.

Maxwell’s equations are solved for the two different regions: inside the waveguide and inside the plasma tube. In the first grid of mesh-grid, the electric and magnetic fields are initialized to zero. Then, using Maxwell’s equations for the electromagnetic wave, with known amplitude and frequency, the values of the electric and magnetic fields in subsequent grids are calculated through the FDTD methods. Figures 9, 10 and 11 shows the temporal variation of the interaction between the RF wave and the plasma switch. This figure illustrate the switching procedure of the wave’s transition for three mentioned cases in Table 1.

This method solves Maxwell’s equations using temporal and spatial approximations at half steps. To accomplish this, the simulation environment is first discretized into a spatial grid, and then, at each time step, the electric and magnetic fields at each grid point are updated. To update the electric and magnetic fields for a wave propagating in the x-direction with polarization in the z-direction, the time-stepping Faraday and Ampère’s laws are applied. Faraday’s Law:

11 $$\begin{aligned} \partial _{t}H_{y}=\dfrac{1}{\mu }[\partial _{x}E_{z}-(M_{source_{y}}+\sigma ^{*}H_{y})]. \end{aligned}$$

Ampere’s law:

12 $$\begin{aligned} \partial _{t}E_{z}=\dfrac{1}{\epsilon }[\partial _{x}H_{y}-(J_{source_{z}}+\sigma E_{z})], \end{aligned}$$

where $E_z$ and $H_y$ are the electric and magnetic fields in the *z*- and *y*-directions, respectively, and perpendicular to the propagation direction (*x*), $m_{\text {source}}$ and $j_{\text {source}}$ represent the magnetic source current and the source current, respectively. Additionally, $\sigma$ and $\sigma ^*$ denote the conductivity and equivalent magnetic losses, respectively. The Yee cube can be rewritten and simplified as follows:

13 $$\begin{aligned} (E_{z}^{n}(i+1)-E_{z}^{n}(i))\left( \dfrac{\Delta t / \Delta x / \mu }{\dfrac{H \Delta t \sigma }{2\mu }+1}\right) +H_{y}^{\dfrac{1}{2}-n}\left( i+\dfrac{1}{2}\right) \left( \dfrac{\dfrac{H \Delta t \sigma }{2\mu }-1}{\dfrac{H \Delta t \sigma }{2\mu }+1}\right) =H_{y}^{\dfrac{1}{2}+n}\left( i+\dfrac{1}{2}\right) , \end{aligned}$$

and

14 $$\begin{aligned} \left( H_{y}^{\dfrac{1}{2}+n}\left( i+\dfrac{1}{2}\right) -H_{y}^{\dfrac{1}{2}n}\left( i-\dfrac{1}{2}\right) \right) \left( \dfrac{\Delta t / \Delta x / \epsilon }{\dfrac{H \Delta t \sigma }{2\epsilon }+1}\right) +E_{z}^{n}(i)\left( \dfrac{\dfrac{ \Delta t \sigma }{2\epsilon }-1}{\dfrac{ \Delta t \sigma }{2\epsilon }+1}\right) =E_{z}^{n+1}(i). \end{aligned}$$

## Discussion

The plasma switch exhibits two functional states: when deactivated (‘off state’), it allows microwave energy to pass through the side arm; when activated (‘on state’), high-density plasma is formed, reflecting incident waves back into the main path, enabling wave amplification or redirection. Unlike solid-state switches, which may suffer from thermal limitations and slower switching speeds, the plasma switch responds within several tens of nanoseconds and supports high-power operation without mechanical contacts. These features make it highly suitable for fast-switching, high-reliability microwave applications.

**Fig. 12:**
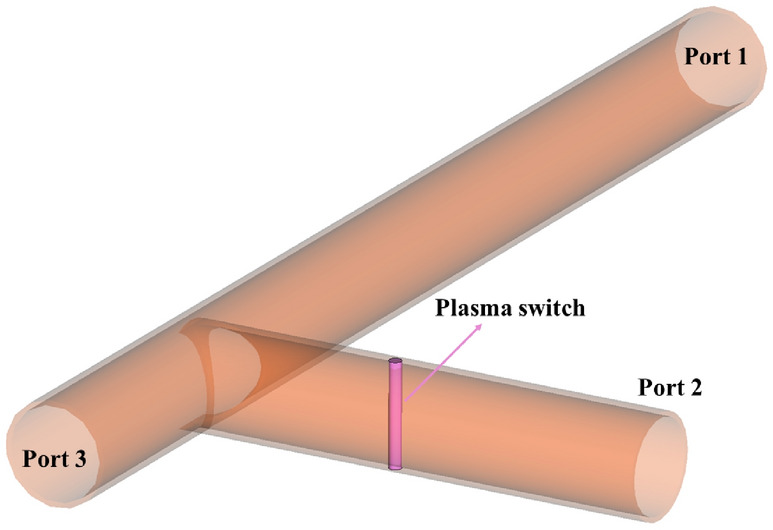
Schematic of a T-shaped circular waveguide in the S-band with three ports: input, side, and output when plasma switch is on.

**Fig. 13:**
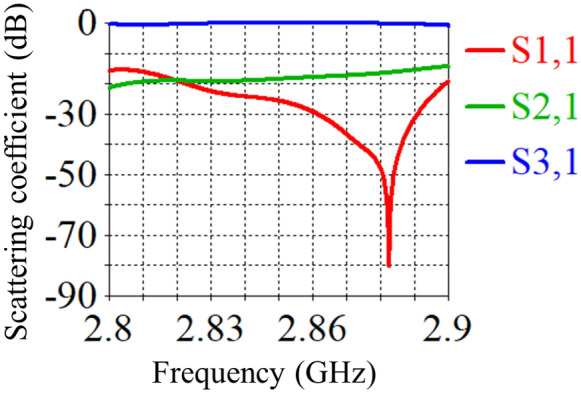
Dispersion coefficient of circular T-shaped waveguide when plasma switch is on.

**Fig. 14:**
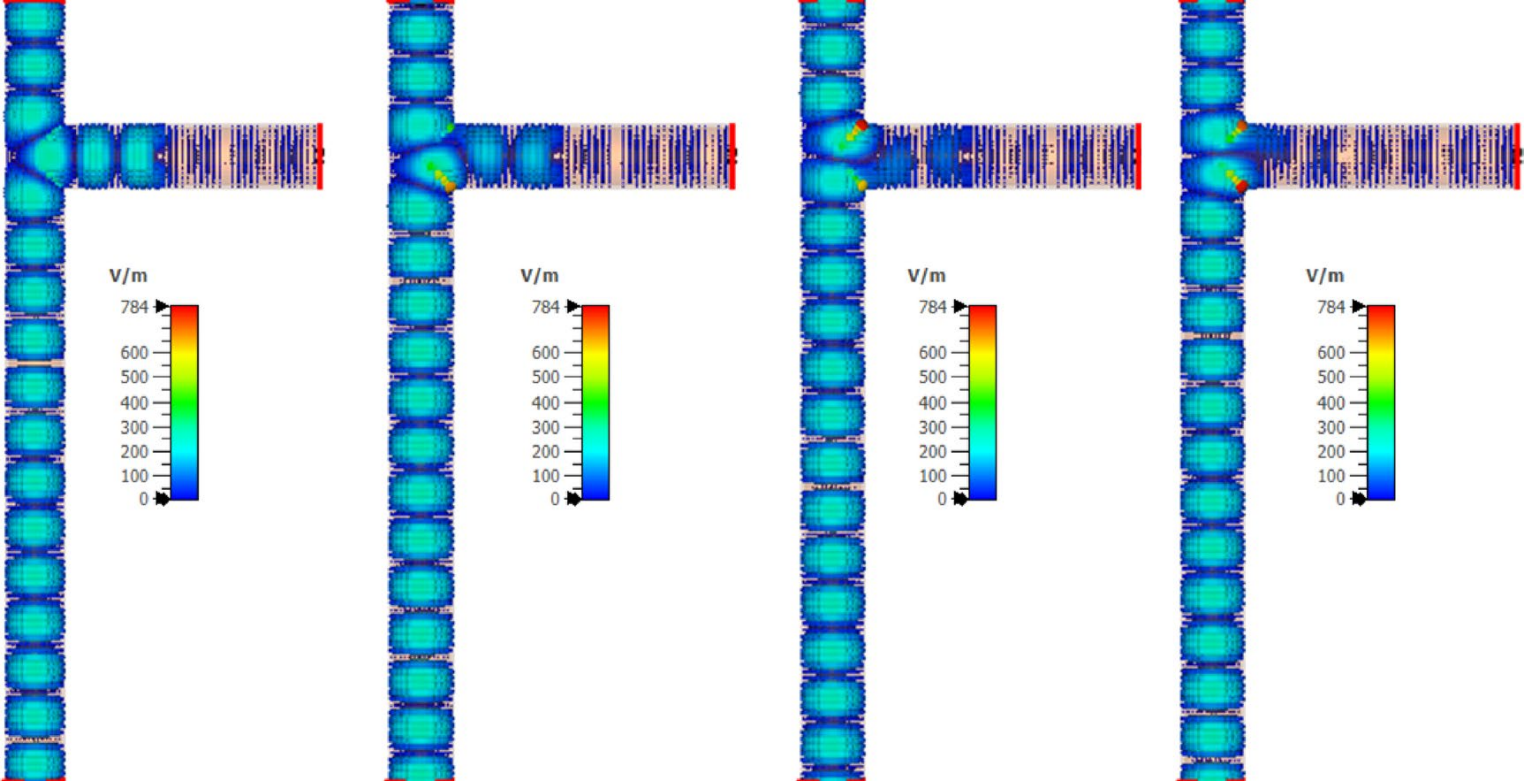
Time variations of the electric field inside the waveguide for the fully reflective case (c).

An electromagnetic wave with a wavelength of 4.5 cm propagates within a circular waveguide and encounters a plasma switch for three cases presented in Table 1. The choice of the tube radius in the plasma switch is based on the phase shift of the incident and reflected waves. The phase shift for the wave after encountering a uniform plasma column with radius *a* is given by $\Delta \phi = \left[ 1 - \sqrt{1 - \frac{\omega _p^2}{\omega ^2}}\right] a$. For the case where ${\omega _p}/{\omega } \ll 1$, the phase shift is purely real, resulting in a zero-degree phase difference. In contrast, for ${\omega _p}/{\omega } \gg 1$, the phase difference combines both real and imaginary components. To adjust the ratio $\omega /c$ at a frequency of 2.86 GHz, the radius *a* must be on the order of $10^{-3}$. Figure 12 shows the complete reflection of the electromagnetic wave from the plasma tube, which occurs due to the wave’s inability to penetrate the plasma at a frequency of 2.86 GHz. Figure 12 present shematic of T-shaped circular waveguide in the S-band when plasma switch is on. Figure 13 compares the scattering coefficients versus frequency for the input wave at all three ports. As observed from this figure, no wave returns to port 1, and the wave that has passed through the plasma switch is negligible (Fig. 14).

**Fig. 15:**
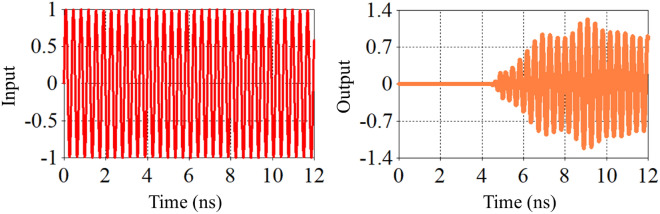
Comparison of the input pulse and the amplitude of the sinusoidal pulse entered from the domain with the compressed pulse exited from the port.

The behavior of the reflected electric field from the switch location is crucial for achieving constructive interference between the reflected wave and the incident wave. The extent of this constructive interference depends on factors such as the tube radius, gas type, gas density, and the placement of the switch. Figure 15 illustrates the temporal evolution of the electric field inside the T-shaped waveguide over time and in the case (c) (complete reflection).

The variations in the electric field near the plasma tube show a good agreement between the numerical solution and the simulation results. As seen in the figure, almost no wave passes through the switch, and the reflected wave exits from port 3 in a clockwise direction. If we consider the input wave from port 1 as a sinusoidal wave with a frequency of 2.86 GHz, a pulse width of 12 nanoseconds, and an amplitude of 1, the output wave from port 3 becomes compressed due to complete reflection from the switch and the creation of a perfectly constructive interference. This compression results in a reduction in pulse width and an increase in wave amplitude. Figure 15 shows an approximate 1.4-fold increase in amplitude. From Fig. 2, it can be seen that the amplitude of the wave entering port 2 without the switch is about 0.6, and the amplitude of the wave exiting port 3 is approximately 0.8. By placing the switch at the appropriate location and reflecting an amplitude of 0.6 from the sidewall and creating constructive interference between these two waves, the amplitude of the output wave increases by a factor of 1.4. Moreover, Fig. 13 illustrates the S-parameter behavior for the plasma switch in the on states, respectively. A comparison of these figures indicates that 2.86 GHz is the operating frequency for this setup. Under varying plasma densities, Fig. 8 shows changes in stored energy, reflecting improved switching efficiency. Furthermore, as demonstrated in Fig. 15a $1.4\times$ increase in output amplitude is observed, which is attributed to constructive interference caused by plasma-induced reflection.

To benchmark our system, we compare its performance with recent state-of-the-art technologies^22,24^. Solid-state switches, while compact, often suffer from thermal limitations and relatively slower response times under high-power conditions. Dielectric-based systems offer good stability but generally lack dynamic tunability. In contrast, our plasma switch exhibits enhanced reflection and waveform modulation capabilities across a range of plasma densities, providing superior adaptability and power handling. These findings highlight the potential of plasma-based solutions for next-generation RF switching and signal amplification applications.

## Conclusion

In this study, we analyzed the behavior of electromagnetic waves within a T-shaped circular waveguide incorporating a plasma switch. The simulations yield key results such as scattering parameters (Figs. 2 and 13), temporal electric field energy distribution (Fig. 8), and output amplitude enhancement due to constructive interference (Figs. 14 and 15). The reported amplitude increase results from passive coherent enhancement through constructive interference. While it does not involve external energy input, it effectively compresses and boosts the output waveform, justifying the term ’amplification’ in the context of signal control.

Based on the simulation results and analyses, the following key points can be highlighted:

Effect of Plasma on Microwave Waves: An increase in plasma density leads to a decrease in the dielectric constant and an enhancement in wave reflection. This indicates that higher plasma densities can effectively reflect waves, significantly improving the performance of waveguide systems.

“Zero Dielectric Constant” Phenomenon: When plasma density becomes substantially high, resulting in the plasma frequency being much greater than the incident wave frequency ($\omega \ll \omega _p$), the dielectric constant approaches zero. This condition causes strong wave reflection from the plasma and prevents the transmission of waves through the plasma.

Impact of Design Parameters: Accurate design of parameters such as plasma density, applied voltage, gas type, and tube dimensions is crucial for achieving optimal performance and enhancing wave reflection. The use of quartz tubes, with their suitable properties, helps maintain plasma density and prevent dispersion.

Future Directions: The findings of this study contribute to a better understanding of the interactions between microwave waves and plasma, and they assist in optimizing plasma switch designs. Future research could focus on more precise simulations and investigating the effects of various operational conditions on plasma switch performance.

Overall, this research sheds light on the complex interactions between electromagnetic waves and plasma, contributing to the development and enhancement of new technologies in this field. Future investigations may focus on refining simulations and exploring the impact of different practical conditions on the performance of plasma switches. However, this study is based on idealized plasma conditions, assuming spatial uniformity and excluding experimental validation. In real-world systems, factors such as plasma non-uniformity, gas dynamics, and electrode configuration may significantly influence device performance. Future research should focus on experimental verification, the development of real-time plasma control techniques, and the incorporation of spatially varying plasma models to enhance the accuracy of predictions and support the practical deployment of plasma-based microwave switching and amplification technologies.

Inaddition, this study indicate that the proposed plasma switch has strong potential for integration into advanced RF architectures that demand rapid and robust signal management. Example applications include defense and weather radar systems, high-power pulse compression modules, and adaptive communication infrastructures in aerospace and satellite systems.

## Data Availability

The datasets generated and/or analyzed during the current study are available from the corresponding author on reasonable request.
